# Circular RNA circPOSTN promotes neovascularization by regulating miR-219a-2-3p/STC1 axis and stimulating the secretion of VEGFA in glioblastoma

**DOI:** 10.1038/s41420-022-01136-9

**Published:** 2022-08-04

**Authors:** Niya Long, Xu Xu, Hongyi Lin, Ying Lv, Shenghui Zou, Han Cao, Xueshu Chen, Yan Zhao, Xiaolan Qi, Hua Yang, Jian Liu, Liangzhao Chu

**Affiliations:** 1 Department of Neurosurgery, the Affiliated Hospital of Guizhou Medical University, Guiyang, Guizhou China; 2 School of Clinical Medicine, Guizhou Medical University, Guiyang, Guizhou China; 3 Department of Clinical Laboratory, Guizhou Cancer Hospital, Guiyang, Guizhou China; 4 Key Laboratory of Endemic and Ethnic Diseases, Ministry of Education & Key Laboratory of Medical Molecular Biology of Guizhou Province, Guizhou Medical University, Guiyang, Guizhou China; 5 Department of Neurosurgery, Guizhou Provincial People’s Hospital, Guiyang, Guizhou China

**Keywords:** Cell growth, Neurological disorders

## Abstract

Glioblastoma (GBM), the most malignant type of astrocytic tumor, is one of the deadliest cancers prevalent in adults. Along with tumor growth, patients with GBM generally suffer from extensive cerebral edema and apparent symptoms of intracranial hyper-pressure. Accumulating evidence has demonstrated that circRNA plays a critically important role in tumorigenesis and progression. However, the biological function and the underlying mechanism of circRNA in GBM remain elusive. In this study, by conducting gene expression detection based on 15 pairs of GBM clinical specimens and the normal adjunct tissues, we observed that circPOSTN showed abnormally higher expression in GBM. Both loss-of-function and gain-of-function biological experiments demonstrated that circPOSTN scheduled the proliferation, migration, and neovascularization abilities of GBM cells. Further, fluorescence in situ hybridization (FISH) assay, quantitative RT-PCR, and subcellular separation suggested that circPOSTN was predominately localized in the cytoplasm and may serve as a competing endogenous RNA (ceRNA). CircRNA-miRNA interaction prediction based on online analytical processing, AGO2-RIP assay, biotin labeled RNA pulldown assay, and dual-luciferase reporter assay revealed that circPOSTN sponged miR-219a-2-3p, limited its biological function, and ultimately upregulated their common downstream gene STC1. Finally, by carrying out in vitro and in vivo functional assays, we uncovered a new regulatory axis circPOSTN/miR-219a-2-3p/STC1 that promoted GBM neovascularization by increasing vascular endothelial growth factor A (VEGFA) secretion. Our study underscores the critical role of circPOSTN in GBM progression, providing a novel insight into GBM anti-tumor therapy.

## Introduction

Glioblastoma (glioblastoma multiforme, GBM) is the most prevalent primary brain cancer originated from star-shaped glial cells, with high aggressiveness and rapid proliferation [1]. Despite the advances in the treatment of GBM, this lethal tumor is highly resistant to anti-tumor therapy, and therefore the prognosis of patients with GBM is extremely dismal [2, 3]. Indeed, from a biological standpoint, GBM cells show numerous pathological changes, such as resistance to cellular apoptosis [4], altered metabolism [5], dysregulated p53 signaling pathway, and incompetent of the immune response. Therefore, an in-depth investigation regarding a novel therapeutic aiming to early recognize GBM patients and improve their clinical outcome, is in urgent need.

Circular RNA (circRNA) is produced by the back-splicing of exons and introns [6]. Due to its distinctive molecular structure, circRNA exerts its biological functions through binding to numerous types of molecules, including RNA, DNA, and protein [7]. Accumulating studies revealed that the aberrant regulation of circRNA expression is at the basis of multiple human disorders, particularly carcinogenesis [8–10]. For example, several circRNAs were identified as gastric cancer (GC)-related circRNAs, and the aberrant expression of them showed strongly interrelated with GC formation and progression [11]. Besides, the regulatory network concerning circRNAs in cervical cancer has drawn great attention. Many of the circRNAs, such as circATP8A2, circ0067934, circ0023404, and circEIF4G2, were demonstrated to function as ceRNA to sponge their target miRNAs and ultimately widely participate in cervical tumor development [12]. In GBM, the importance of circRNAs has already been validated by several investigators. As novel biomarkers, circFOXO3, circ_0029426, and circ-SHPRH were combined as a prognostic model for early diagnosis and prognosis of GBM patients [13]. Based on circRNA deep sequencing and mass spectrometer technology, Yibing Yang et al. identified a circFBXW7 that encoded a novel protein, termed FBXW7-185aa, which suppressed GBM proliferation and caused cell cycle arrest [14]. Despite accumulating and exciting advances in circRNA tumor biology, the exact molecular functions involved in circRNA biogenesis and the potential for circRNA to act as an anti-tumor therapeutic target is not yet clear.

In the current research, our objective was to explore the biological role of circPOSTN in GBM. By comparing the expression pattern of circPOSTN in 15 pairs of GBM tissues and the normal adjunct tissues, we identified that circPOSTN was typically overexpressed in GBM. Functional experiments revealed that circPOSTN enhanced GBM cell proliferation, migration, and neovascularization capacities. Mechanically, circPOSTN exerted its tumor-promoting effect by acting as a ceRNA to inhibit the miR-219a-2-3p/STC1 axis, upregulating the expression of oncogene STC1, and therefore facilitated VEGFA secretion.

## Results

### CircPOSTN is highly expressed in GBM and mainly located in the cytoplasm

**Fig. 1:**
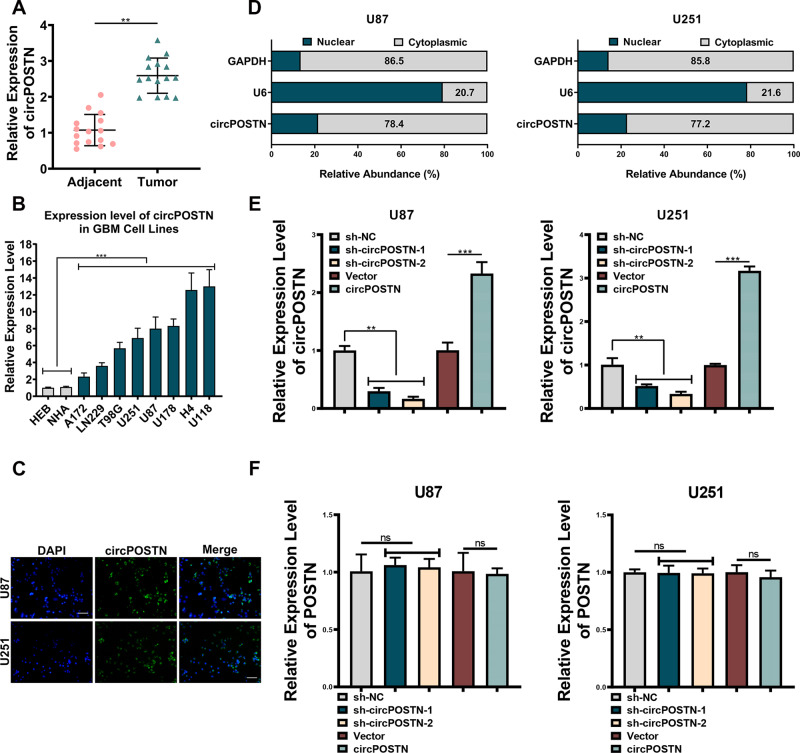
CircPOSTN is overexpressed in glioblastoma (GBM) and is mainly located in the cytoplasm. **A** The expression level of circPOSTN was determined by quantitative RT-PCR in 15 pairs pf primary GBM tumors and adjacent tissues. **B** Quantitative RT-PCR analysis of circPOSTN expression in eight GBM cell lines (A172, LN2229, T98G, U251, U87, U178, H4, and U118), one normal human astrocyte cell line (NHA), and one human normal glial cell line (HEBs). **C** The subcellular location of circPOSTN was detected by fluorescent in situ hybridization (FISH). circPOSTN was stained green, and the nuclei were stained blue. **D** The cytoplasmic and nuclear fractions of U87 and U251 cells were separated. Then, qPCR analysis was used to determine the subcellular location of circPOSTN. U6: nuclear control; GAPDH: cytoplasmic control. The expression of circPOSTN (**E**) and POSTN (**F**) was measured by qPCR after circPOSTN knockdown (left panel) and overexpression (right panel) in U87 and U251 cell lines. Data are presented as the mean ± SD. **P < 0.01, ***P < 0.001, ns not significant.

Before exploring the biological functions of circPOSTN, we first detected the expression level of circPOSTN in GBM. Quantitative RT-PCR analysis showed that the expression levels of circPOSTN in GBM tissues were 2.5-fold higher than in the adjacent normal tissues (Fig. 1A, *P* < 0.01). Moreover, the elevated expression level of circPOSTN was observed in GBM cell lines compared with the normal human astrocyte cell line (NHA) and the normal human glial cell line (HEB; Fig. 1B, all *P* < 0.01). These results indicated that circPOSTN might exert its cancer-promoting effect in GBM.

To explore the possible mechanism underlying the effects of circPOSTN in GBM, we applied FISH assays to detected the subcellular location of circPOSTN. As shown in Fig. 1C, circPOSTN was mainly localized in the cytoplasm and was weakly in the nucleus. For further verification of this finding, we extracted the total RNA from the nuclear and cytoplasmic fractions of U87 and U251 cells, respectively. Quantitative RT-PCR analysis further confirmed that circPOSTN was primarily localized in the cytoplasmic compartment (Fig. 1D, 78.4% in U87 cells and 77.2% in U251 cells).

We then knockdown the expression level of circPOSTN using two independent shRNAs constructs (sh-circPOSTN-1 and sh-circPOSTN-2) to explore the biological functions of circPOSTN in GBM. The knockdown efficiency of shRNAs was determined by quantitative RT-PCR (Fig. 1E, left panel, all *P* < 0.01) compared to the scrambled shRNA control. Similarly, we used the overexpression plasmids to upregulate circPOSTN expression in GBM cells, which successfully boosted a 2~3-fold greater expression of circPOSTN (Fig. 1E, right panel, *P* < 0.01). Since circular RNA is transcribed from the same gene position with the mRNA [15], we detected the expression levels of liner transcripts POSTN under shRNAs and overexpressing plasmids inference. Quantitative RT-PCR confirmed that either shRNAs or overexpressing constructs of circPOSTN had no effects on POSTN expressions (Fig. 1F).

### Overexpression of circPOSTN boosts GBM cell proliferation, migration, and neovascularization

**Fig. 2:**
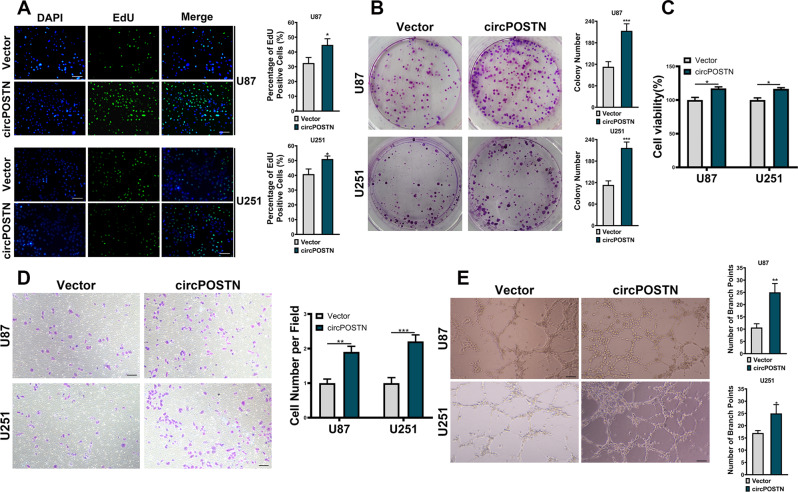
Upregulation of circPOSTN improves GBM cell proliferation, migration, and tumor angiogenesis in vitro. **A** Cell proliferation ability of GBM cells with or without circPOSTN overexpressing was determined by EdU staining (green). The Edu-positive cells were recorded and quantified. The nuclei were stained with DAPI (blue). **B** The clone formation assay was used to assess the clone formation capacity of GBM cells with or without circPOSTN overexpressing. **C–E** The human umbilical vein endothelial cells (HUVECs) were cultured with conditioned media from GBM cells with or without circPOSTN overexpressing. **C** The viability of HUVEC was evaluated by MTT assay. **D** The transwell migration assay was used to evaluate the migration ability of HUVECs. **E** The tubule formation assay was used to assess the angiogenesis ability of GBM cells. The tubule formation ability of HUVEC was analyzed by measuring the branch points and tubule number. Data are presented as the mean ± SD. *P < 0.05, **P < 0.01, ***P < 0.001.

To date, the exact biological function of circPOSTN in GBM remains elusive. Therefore, we characterized it in GBM cells. We initially incorporated the U87 and U251 cells from both groups (empty vector and circPOSTN overexpression) with EdU. Quantification of EdU-positive cells revealed that circPOSTN overexpression apparently promoted the proliferation capacity of GBM cells (Fig. 2A, *P* < 0.05). Similar results were observed from the clone formation assay, which showed the much more clones formed after circPOSTN upregulation (Fig. 2B, *P* < 0.001). To determine whether circPOSTN exerts its tumor-promoting effect by inducing neovascularization, we conducted the functional experiments using human umbilical vein endothelial cells (HUVECs), which were cultured in the conditional medium from U87 and U251 cells. MTT assays suggested that overexpression of circPOSTN had the proliferation-promoting effect on HUVECs (Fig. 2C, *P* < 0.05). Transwell migration assays indicated that circPOSTN expression was involved in the cellular migration of HUVECs (Fig. 2D, *P* < 0.01). Moreover, we used in vitro tubular formation assays to functionally analyze the effects of circPOSTN in GBM. As shown in Fig. 2E, the conditional medium from GBM cells with circPOSTN overexpressing enhanced the angiogenesis ability of HUVECs to a large extent in vitro (*P* < 0.05). The above results supported the idea that upregulation of circPOSTN expression enhanced GBM cell proliferation, migration, and neovascularization in vitro.

### The silencing of circPOSTN inhibits the proliferation, migrations and neovascularization abilities of GBM cells

**Fig. 3:**
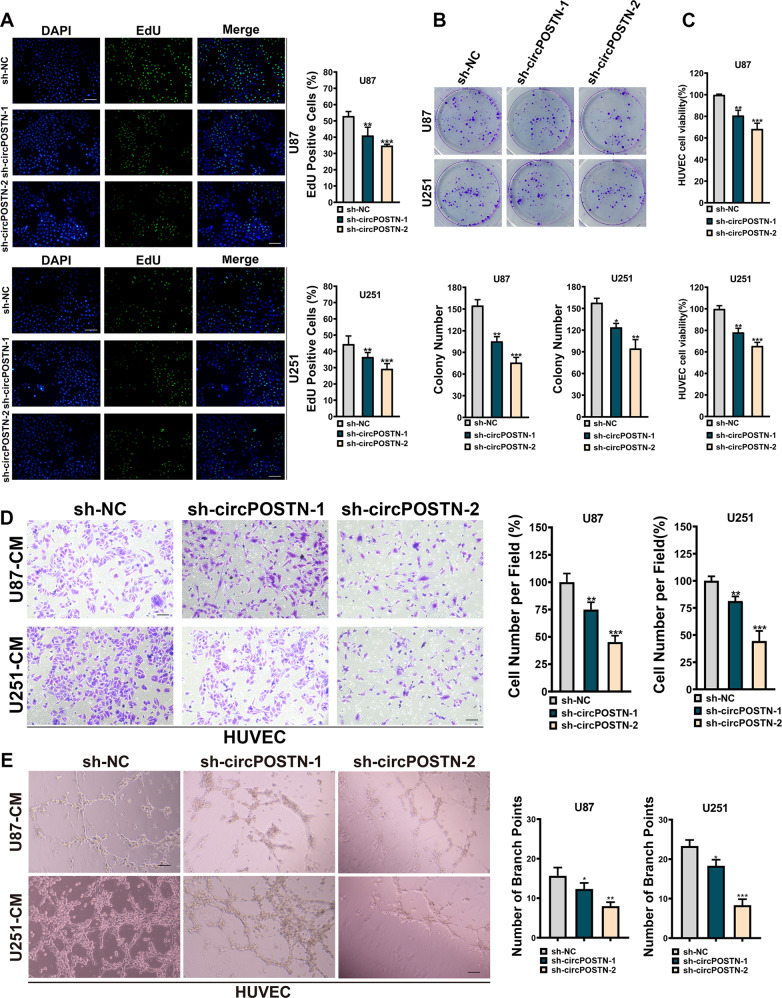
Interference of circPOSTN inhibits proliferation, migration, and tumor angiogenesis abilities of GBM in vitro. **A** EdU staining was used to measure the proliferation ability of GBM cells (U87 and U251) after circPOSTN knockdown with two individual shRNAs (sh-circPOSTN-1 and sh-circPOSTN-2). **B** The influence of circPOSTN silencing on clone formation ability of GBM cells was assessed using clone formation assay. **C–E** The HUVECs were cultured in the conditional medium from U87 and U251 cells transfected with sh-NC, sh-circPOSTN-1, or sh-circPOSTN-2. **C** The effect of circPOSTN knockdown on HUVECs proliferation was detected by MTT assay. **D** Transwell migration assays were performed to determine the migration ability of HUVECs. **E** The effect of circPOSTN interference on tubule formation of HUVECs was measured by tubule formation assay. The quantification of tubule formation was defined based on the branch points and tube numbers. Data are presented as the mean ± SD. *P < 0.05, **P < 0.01, ***P < 0.001.

For a better illustration of the tumor-promoting effects of circPOSTN in GBM, we knocked down circPOSTN expressions by using two independent shRNAs targeting circPOSTN (Fig. 1E). Not surprisingly, both EdU incorporation assays and clone formation assays confirmed that interference of circPOSTN expression significantly impaired the proliferation ability of GBM cells (Fig. 3A, B, all *P* < 0.01). Similarly, we cultured the HUVECs in conditional medium from GBM cells with circPOSTN silenced. The opposite phenotypes were obtained compared with circPOSTN overexpressing results. As shown in Fig. 3C, depletion of circPOSTN expression inhibited the proliferation-promoting effect of GBM cells in HUVECs. Transwell migration assays illustrated that silenced circPOSTN dramatically suppressed the migration ability of HUVECs (Fig. 3D, all *P* < 0.01). Furthermore, tubule formation assays showed that knockdown of circPOSTN apparently impaired the positive effect of GBM cells on the angiogenic tube formation ability of HUVECs (Fig. 3E, all *P* < 0.05). In conclusion, inhibiting circPOSTN expression suggests the loss of tumor-promoting function of GBM cells in vitro.

### CircPOSTN exerts pro-oncogenic functions by acting as a ceRNA to sponge miR-219a-2-3p

**Fig. 4:**
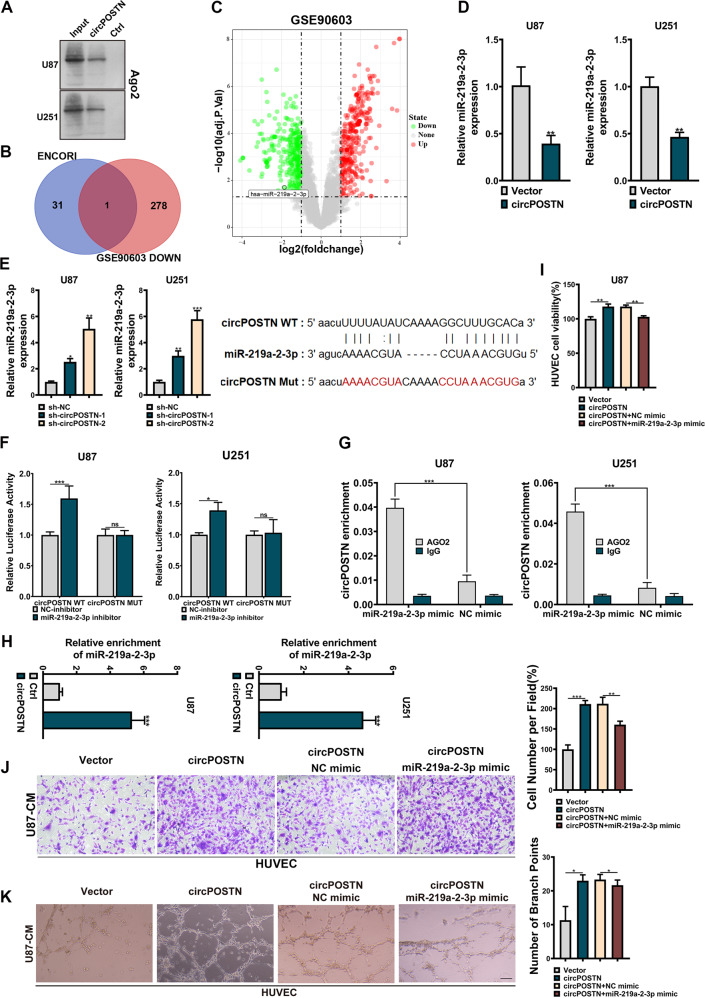
circPOSTN acts as a ceRNA to sponge miR-219a-2-3p. **A** Western blotting analysis showed the endogenous AGO2 proteins were pulldown by circPOSTN in U87 and U251 cells. **B** The intersection of miRNAs that were predicted to be sponged by circPOSTN (*n* = 32) based on the ENCORI database and were downregulated (*n* = 279) in GBM tissues based on the GEO dataset (GSE90603). The has-miR-219a-2-3p was found. **C**. The volcano plot for differently expressed miRNAs in GBM tissues compared with its adjacent tissues based on the GSE90603 dataset. The position of has-miR-219a-2-3p was indicated. Quantitative RT-PCR was used to determine the expression level of miR-219a-2-3p in U87 and U251 with circPOSTN overexpressed (**D**) or knockdown (**E**). **F** The binding sequence of miR-219a-2-3p and circPOSTN (circPOSTN WT) and its mutation sequence (circPOSTN Mut) were cloned into the luciferase reporter vectors. The dual-luciferase reporter assay was used to detect the luciferase activity in cells co-transfected with circPOSTN WT/Mut and miR-219a-2-3p inhibitor/control. **G** The RIP experiments using anti-AGO2 or anti-normal IgG antibodies were carried out to investigate the interaction of circPOSTN and miR-219a-2-3p in cells with circPOSTN overexpressed. **H** The biotinylated circPOSTN was used to conduct RNA pulldown followed by qPCR to test the level of miR-219a-2-3p in U87 and U251 cells. **I–K** The HUVECs were cultured in the medium extracted from U87 cells with circPOSTN or miR-219a-2-3p overexpressed. **I** MTT assays were performed to determine the proliferation ability of HUVECs. **J** Transwell migration assays were carried out to assess the migration ability of HUVECs. **K** The tubule formation assays were used to analyze the impact of circPOSTN or miR-219a-2-3p on tubule formation of HUVECs. Data are presented as the mean ± SD. *P < 0.05, **P < 0.01, ***P < 0.001.

As previously described in Fig. 1C, D, circPOSTN is predominantly located in the cytoplasm, implying that it may exert its oncogenic function by acting as a competing endogenous RNA (ceRNA) to sponge its target miRNAs [16]. To verify this hypothesis, we initially conducted RNA pulldown assays using biotinylated circPOSTN followed by western blotting analysis. Immunoblotting using AGO2 antibodies revealed that circPOSTN was inclined to be involved in the RNA-induced silencing complex (RISC; Fig. 4A). To search the miRNAs that were absorbed in the circPOSTN-medicated RISC, we first predicted the miRNAs that could interact with circPOSTN using an online tool ENCORI (http://starbase.sysu.edu.cn/index.php), in which 32 miRNAs were identified. Meanwhile, by analyzing the miRNA expression profile from the GEO dataset (GSE90603), we found 279 miRNAs that showed downregulated in the GBM tissues. Taking the intersection of these predicted miRNAs, we found miR-219a-2-3p to be the potential target miRNA of circPOSTN (Fig. 4B, C).

For verification, we initially overexpressed circPOSTN in U87 and U251 cells, and correspondingly, we found the expression level of miR-219a-2-3p reduced to a large extent (Fig. 4D, *P* < 0.01). In contrast, knockdown of circPOSTN promoted the expression of miR-219a-2-3p in GBM cells (Fig. 4E, *P* < 0.01). Following this, we constructed a dual-luciferase reporter system, in which the sequences on circPOSTN that were predicted to interact with miR-219a-2-3p were cloned in the pmirGLO vectors (circPOSTN WT). Correspondingly, the mutant sequence was used as a negative control (circPOSTN Mut). As shown in Fig. 4F, downregulating of miR-219a-2-3p apparently increased the luciferase activity of circPOSTN WT but had no impact on the luciferase activity of circPOSTN Mut. RNA immunoprecipitation (RIP) assay using the AGO2 antibodies revealed that overexpression of miR-219a-2-3p promoted the enrichment of circPOSTN in the RISC compared to the negative control group (Fig. 4G, all *P* < 0.001). Finally, we labeled the circPOSTN with biotin and conducted RNA pulldown experiments. Quantitative RT-PCR found a significant enrichment of miR-219a-2-3p from the RNA precipitated by biotin labeled circPOSTN (Fig. 4H, all *P* < 0.001). To sum up, these results pointed out that miR-219a-2-3p is one of the major downstream miRNAs of circPOSTN in GBM.

Based on the above results, we conducted a series of functional rescue experiments using the HUVECs as mentioned above. Not surprisingly, the proliferation, migration, and neovascularization abilities of HUVECs were dramatically promoted by circPOSTN overexpression. However, these phenotypes could be partially rescued by exogenously add-back of miR-219a-2-3p (Fig. 4I–K; Supplementary Fig. S1A, B). To sum up, these findings revealed that circPOSTN promotes GBM neovascularization by sponging miR-219a-2-3p.

### CircPOSTN promotes GBM neovascularization through miR-219a-2-3p/STC1 axis

**Fig. 5:**
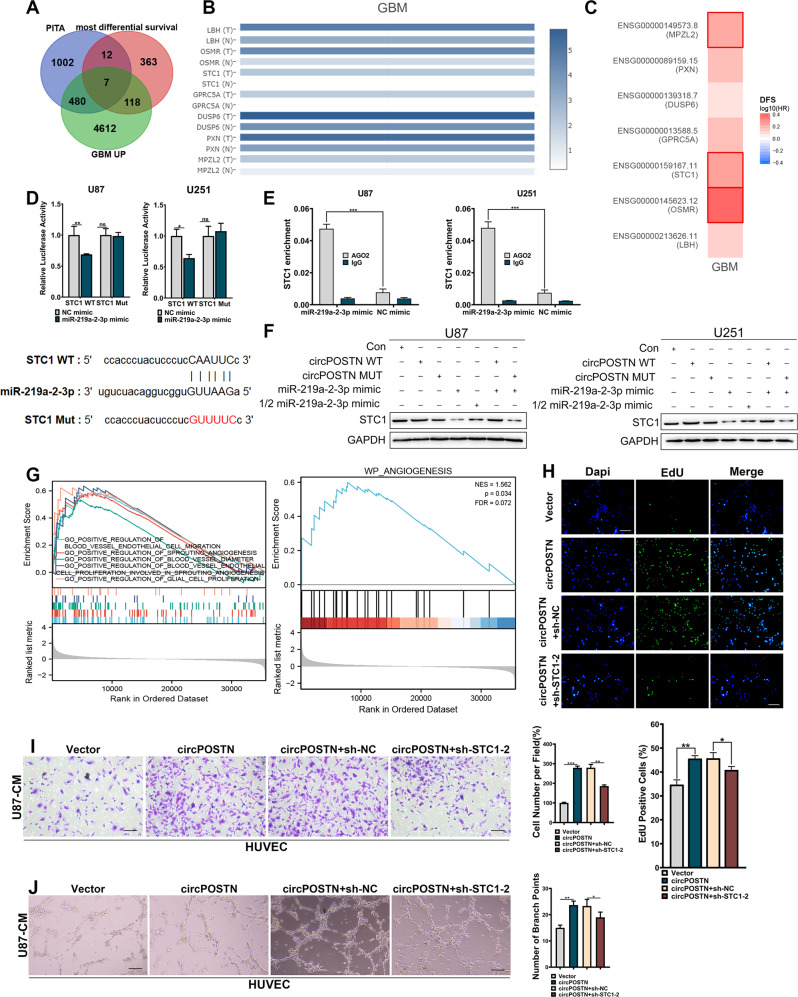
CircPOSTN acts as a ceRNA and binds with miR-219a-2-3p to upregulate STC1 expression. **A** The intersection of genes predicted to: i) binds with miR-219a-2-3p based on an online tool PITA (https://genie.weizmann.ac.il/pubs/mir07/mir07_dyn_data.html), *n* = 1501; ii) be the most differential survival genes in GBM tissues based on GEPIA2 database, *n* = 500; iii) be upregulated in GBM tissues based on GEPIA2 database, *n* = 5217. Seven genes (LBH, OSMR, STC1, GPRC5A, DUSP6, PXN, and MPZL2) were identified. **B** The expression levels of these seven genes in GBM were analyzed based on the TCGA dataset. **C** The disease free survival (DFS) analysis of the relationship between these seven genes and GBM survival prognosis based on GEPIA2 database. **D** The sequence in the STC1 gene predicted to bind with miR-219a-2-3p was cloned into the luciferase reporter vectors (STC1 WT). The region-mutant sequence was used as control (STC1 Mut). Then, the dual-luciferase reporter assays were conducted to examine the relative luciferase activity of U87 and U251 cells co-transfected with STC1 WT-Mut vectors and miR-219a-2-3p mimic/control. **E** The expression level of STC1 was precipitated by anti-AGO2 antibodies using RIP assays. The U87 and U251 with miR-219a-2-3p overexpressed were used. **F** Western blotting analysis of STC1 in U87 and U251 cells co-transfected with STC1 WT-Mut vectors and miR-219a-2-3p mimic/control. The GAPDH was used as endogenous control. **G** GSEA results based on the TCGA dataset indicated that the high expression of STC1 was significantly correlated with the neovascularization and cell proliferation abilities of GBM. **H–J** The HUVECs were cultured in the conditioned medium from U87 cells with circPOSTN overexpressed with/without STC1 silenced. **H** Edu staining assay was used for measuring the proliferation ability of HUVECs. **I** The migration ability of HUVECs was detected by transwell migration assays. **J** The effects of circPOSTN and STC1 on the neovascularization ability of U87 cells were determined by tubule formation assays. Data are presented as the mean ± SD. *P < 0.05, **P < 0.01, ***P < 0.001. NS not significant.

To identify the shared downstream involved in the circPOSTN/miR-219a-2-3p axis, we found 1501 potential target genes of miR-219a-2-3p using an online tool PITA (http://genie.weizmann.ac.il/pubs/mir07/mir07_dyn_data.html), which predicts the target mRNAs of the specific miRNAs using the target-site accessibility and free energy prediction algorithm [17]. Meanwhile, based on the GEPIA2 database, we found the top 500 genes that were most correlated survival outcomes of GBM patients and 5217 genes that were upregulated in GBM tissues. The intersection of all three gene sets identifies seven potential target genes (including LBH, OSMR, STC1, GPRC5A, DUSP6, PXN, and MPZL2) absorbed in the circPOSTN/miR-219a-2-3p axis (Fig. 5A). GEPIA2 database showed the expression levels of these seven genes according to the TCGA dataset (Fig. 5B). Disease free survival analysis revealed that among these seven highly expressed genes in GBM, three genes (MPZL2, STC1, and OSMR) showed significantly associated with the prognosis of GBM patients (Fig. 5C, Table 1). Moreover, univariate Cox regression analysis based on TCGA GBM cohort revealed that the expression level of STC1 was correlated to the overall survival of GBM patients (HR = 1.656, *P* = 0.005; Table 2). Multivariate survival analysis was also conducted to confirm that the expression level of STC1 was an independent prognostic factor for patients with GBM (HR = 1.492, *P* = 0.029; Table 2). Besides, stratification analysis showed that STC1 was not associated with subtype of GBM patients (Table 3).

**Table 1:** Clinicopathological characteristics of patient samples from the TCGA GBM cohort.

Characteristic	levels	Overall
*n*		168
Gender, *n* (%)	Female	59 (35.1%)
	Male	109 (64.9%)
Race, *n* (%)	Asian	5 (3%)
	Black or African American	11 (6.6%)
	White	150 (90.4%)
Karnofsky performance score, *n* (%)	<80	36 (28.1%)
	≥80	92 (71.9%)
IDH status, *n* (%)	WT	149 (92.5%)
	Mut	12 (7.5%)
OS event, *n* (%)	Alive	32 (19%)
	Dead	136 (81%)
DSS event, *n* (%)	Alive	34 (21.9%)
	Dead	121 (78.1%)
PFI event, *n* (%)	Alive	32 (19%)
	Dead	136 (81%)
Age, median (IQR)		60 (50.75, 69)

**Table 2:** Univariate and multivariate analyses of various prognostic parameters in patients from the TCGA GBM cohort.

Characteristics	Total(N)	Univariate analysis		Multivariate analysis	
		Hazard ratio (95% CI)	*P* value	Hazard ratio (95% CI)	*P* value
Gender (Male vs. Female)	168	1.026 (0.719–1.466)	0.887		
Race (White&Black or African American vs. Asian)	166	1.500 (0.476–4.723)	0.488		
Age (>60 vs. ≤60)	168	1.365 (0.973–1.915)	0.072	1.109 (0.776–1.585)	0.571
Karnofsky performance score (≥80 vs. <80)	128	0.838 (0.538–1.305)	0.434		
IDH status (Mut vs. WT)	161	0.301 (0.138–0.654)	**0.002**	0.363 (0.160–0.821)	**0.015**
STC1 (High vs. Low)	168	1.656 (1.165–2.353)	**0.005**	1.492 (1.043–2.136)	**0.029**

*p* values that are statistically significant are shown in bold.

**Table 3:** Correlation between STC1 expression and clinicopathologic characteristics of GBM patients.

Characteristic	Low expression of STC1	High expression of STC1	*p*
*n*	84	84	
Gender, *n* (%)			1.000
Female	30 (17.9%)	29 (17.3%)	
Male	54 (32.1%)	55 (32.7%)	
Race, *n* (%)			0.501
Asian	1 (0.6%)	4 (2.4%)	
Black or African American	6 (3.6%)	5 (3%)	
White	75 (45.2%)	75 (45.2%)	
Karnofsky performance score, *n* (%)			0.632
<80	20 (15.6%)	16 (12.5%)	
≥80	45 (35.2%)	47 (36.7%)	
IDH status, *n* (%)			0.128
WT	71 (44.1%)	78 (48.4%)	
Mut	9 (5.6%)	3 (1.9%)	
OS event, *n* (%)			0.556
Alive	14 (8.3%)	18 (10.7%)	
Dead	70 (41.7%)	66 (39.3%)	
DSS event, *n* (%)			0.589
Alive	15 (9.7%)	19 (12.3%)	
Dead	62 (40%)	59 (38.1%)	
PFI event, *n* (%)			0.844
Alive	17 (10.1%)	15 (8.9%)	
Dead	67 (39.9%)	69 (41.1%)	
Age, mean ± SD	59.18 ± 14.22	59.26 ± 12.87	0.968

To further confirm the downstream target, we conduct luciferase reporter assays using the constructs containing the binding sequences (STC1 WT) and mutated sequences (STC1 Mut) on STC1. As shown in Fig. 5D, elevated miR-219a-2-3p expression dramatically downregulated the luciferase intensity of STC1 WT, while the STC1 Mut remained unaffected. RIP assay using the AGO2 antibodies indicated that overexpression of miR-219a-2-3p contributed to the enrichment of STC1 mRNA in RISC compared with the negative control (Fig. 5E, *P* < 0.001). Furthermore, western blotting analysis suggested that overexpression of circPOSTN (circPOSTN WT), but not the mutated circPOSTN (circPOSTN Mut), apparently upregulated STC1 protein level. Even further, such phenomenon was reversed by exogenous overexpression of miR-219a-2-3p. And the more obvious its reversal effect was found when more miR-219a-2-3p was added (Fig. 5F). In summary, these results demonstrated that circPOSTN acts as a ceRNA to sponge miR-219a-2-3p and upregulates STC1 in GBM.

To confirm the regulatory effects of circPOSTN/miR-219a-2-3p/STC1 axis in GBM progression, we conducted gene set enrichment analysis (GSEA) using TCGA dataset from GBM samples and found that elevated STC1 expression linked to the neovascularization and GBM cell proliferation phenotypes (Fig. 5G, all *P* < 0.05). For verification, we performed in vitro functional rescue experiments using the HUVECs cultured in the conditional medium from GBM cells co-transfected with circPOSTN overexpressing vectors and sh-STC1. The empty vectors and sh-NC were used as the negative control, respectively. As shown in Fig. 5H–J and Supplementary Fig. S2, a high level of circPOSTN significantly boosted the proliferation, migration, and neovascularization abilities of HUVECs, which were partially reversed due to the inhibition of downstream STC1 expression.

### CircPOSTN promotes GBM neovascularization by increasing the secretion of VEGF

**Fig. 6:**
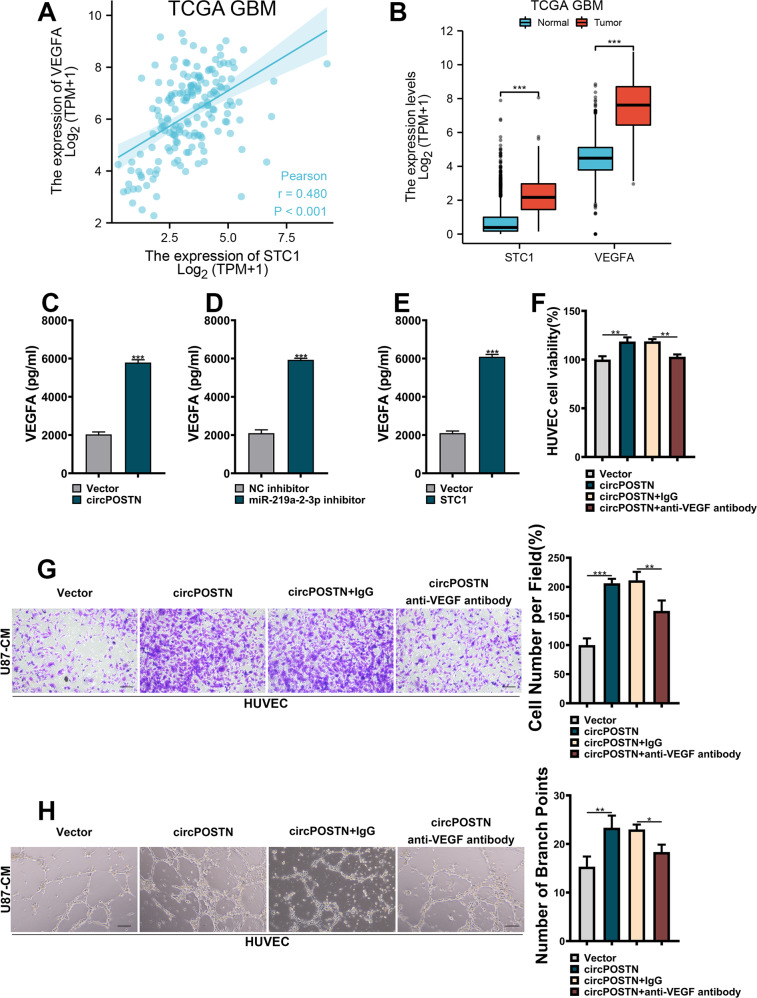
CircPOSTN upregulates STC1 and promotes GBM proliferation, migration, and neovascularization. **A** The expression association between STC1 and VEGFA was analyzed based on the TCGA GBM cohort. **B** The expression levels of STC1 and VEGFA were obtained from the TCGA GBM cohort. **C–E** The VEGFA levels from the culture medium of U87 cells were detected by ELISA. U87 cells were transfected with circPOSTN overexpressing vectors (**C**), miR-219a-2-3p inhibitors (**D**), STC1 overexpressing vectors (**E**), or the corresponding controls. **F–H** The U87 cells were transfected with circPOSTN overexpressing or empty control vectors. Then, the HUVECs were cultured in the conditioned medium from U87 cells incubated with anti-VEGF or anti-IgG antibodies. **F** The MTT measurement of cell proliferation. **G** The transwell migration assays were conducted to determine the cell migration capacity. **H** The tubule formation assays showing the pro-angiogenic effects of U87 cells. Data are presented as the mean ± SD. *P < 0.05, **P < 0.01, ***P < 0.001. NS not significant.

Since the effects of STC1 in GBM neovascularization remains unclear, we then wondered if circPOSTN/miR-219a-2-3p/STC1 axis promoted GBM neovascularization by upregulating vascular endothelial growth factor A (VEGFA) expression. Co-expression analysis was carried out using the TCGA dataset from GBM tissues, and a positive correlation was found between the expression levels of STC1 and VEGFA (Fig. 6A, Person’s r = 0.480, *P* < 0.001). Based on this result, we further observed that both STC1 and VEGFA showed highly expressed in GBM tissues compared to the normal adjunct tissues (Fig. 6B). As expected, we found the increased VEGFA secretion in the culture medium from circPOSTN overexpressing cells using ELISA assays (Fig. 6C, *P* < 0.001). The similar results were observed when perturbating the expression of miR-219a-2-3p (Fig. 6D, *P* < 0.001) or overexpressing the STC1 (Fig. 6E, *P* < 0.001). More importantly, we generated four kinds of conditional medium, including the culture medium from negative control group (Vector-CM), circPOSTN overexpression group (circPOSTN-CM), circPOSTN-CM incubated with normal IgG antibodies group (circPOSTN+IgG-CM), and circPOSTN-CM incubated with VEGFA antibodies group (circPOSTN+aVEGFA-CM). Then, we carried out in vitro functional experiments by culturing HUVECs in these four kinds of conditional mediums. Expectedly, the HUVECs cultured in the circPOSTN-CM and circPOSTN+IgG-CM showed stronger proliferation, migration, and neovascularization abilities, which could be rescued by adding VEGFA antibodies in the conditional medium (Fig. 6F–H). To sum up, circPOSTN promotes GBM cell neovascularization by sponging miR-219a-2-3p, upregulating STC1 expression, and ultimately promoting the secretion of VEGFA.

### CircPOSTN/miR-219a-2-3p/STC1 axis promotes GBM tumor formation in vivo

**Fig. 7:**
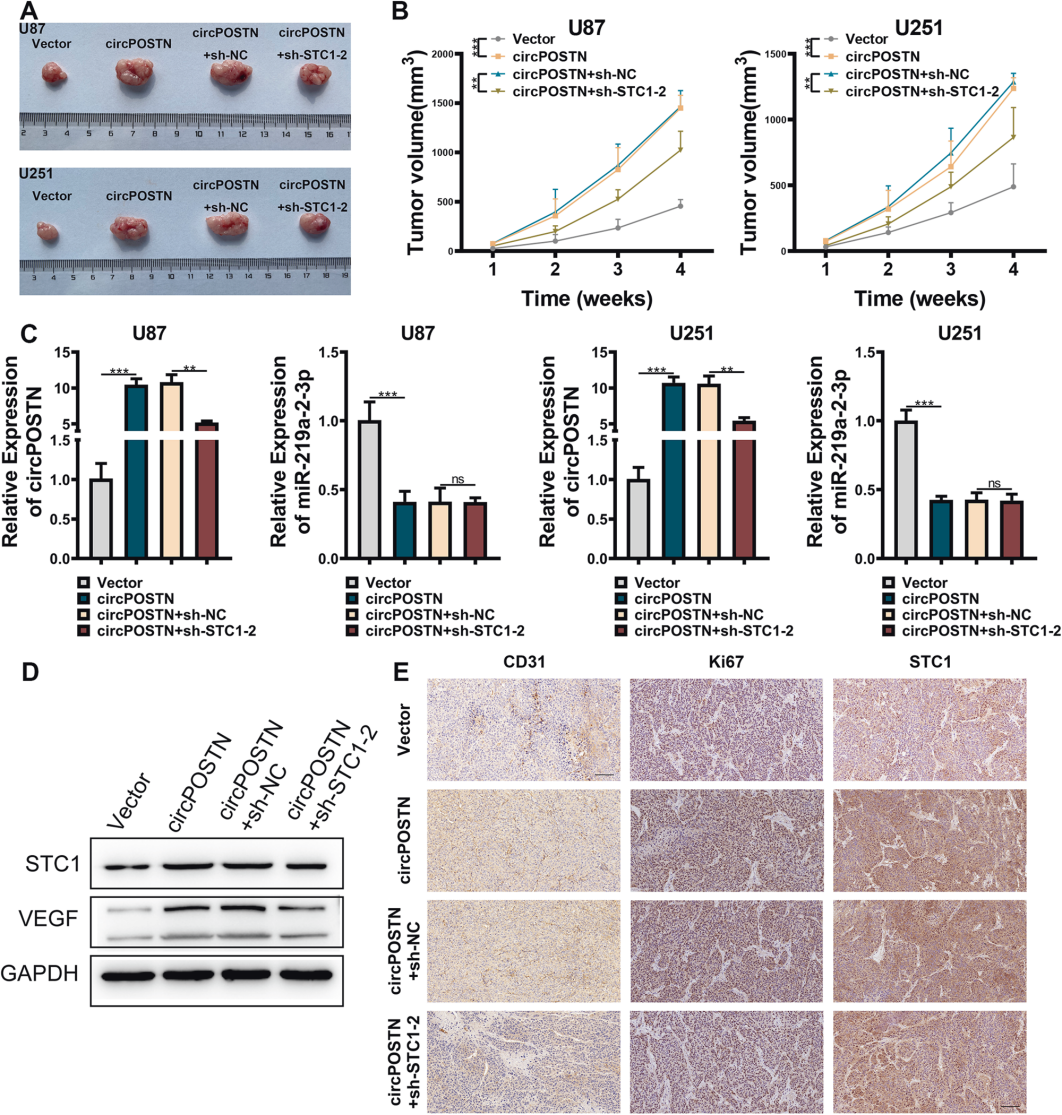
The overexpression of circPOSTN boosts GBM tumorigenesis in vivo. **A–E** The U87 and U251 cells were stably overexpressed with the circPOSTN. Then, the cells were stably transfected with shRNAs targeting the STC1 gene or the negative control (sh-NC). The nude mice were randomly divided into four groups: Negative control (Vector), circPOSTN overexpression (circPOSTN), circPOSTN overexpression with sh-control (circPOSTN + sh-NC), and circPOSTN overexpression with STC1 silenced (circPOSTN overexpression + sh-STC1-2). The U87 and U251 cells were subcutaneously implanted into the nude mice to generate the xenograft tumor models. **A** The representative images of subcutaneous xenograft tumors stripped from nude mice. **B** The growth curve of subcutaneous tumors was plotted by measuring the tumor volume every week. **C** Quantitative RT-PCR was used to analyze the expression levels of circPOSTN and miR-219a-2-3p from xenograft tumors. **D** Western blotting analysis for the expression levels of STC1 and VEGF proteins of subcutaneous tumors. GAPDH was used as endogenous control. **E** The representative images of IHC staining for STC1, Ki-67, and CD31 in subcutaneous tumors. Data are presented as the mean ± SD. **P < 0.01, ***P < 0.001. NS not significant.

Next, we sought to confirm the tumor-promoting effect of circPOSTN/miR-219a-2-3p/STC1 axis in vivo using the xenograft tumor model. U87 and U251 cells were stably overexpressed with circPOSTN and stably silenced STC1 expression. The empty vectors and sh-NC were used as the negative controls, respectively. Then, the cells were subcutaneously injected into the left flank side nude mice. After a four-week feeding period, larger xenograft tumors and faster growth rates were found in the circPOSTN overexpressing group. However, the xenograft tumors with both circPOSTN overexpressing and STC1 silencing showed no significant differences in size and in growth rate (Fig. 7A, B). Quantitative RT-PCR analysis showed that the expression of miR-219a-2-3p was downregulated with increasing circPOSTN expression in both U87 and U251 cells (Fig. 7C). Furthermore, we performed western blotting analysis using proteins extracted from the xenograft tumors and found the increased STC1 and VEGFA expressions in circPOSTN overexpressing tumors compared to the control tumors, while tumors with both circPOSTN overexpressed and STC1 silenced showed no such difference (Fig. 7D). Finally, we used the paraffin sections made from xenograft tumors and conducted IHC staining with CD31 (staining vessels), Ki67 (representing proliferation), and STC1 antibodies. As indicated in Fig. 7E, the stronger CD31, Ki67, and STC1 immunostainings were found in the circPOSTN overexpressing groups, while no significant differences were identified in the circPOSTN overexpressing and STC1 silencing group. In summary, these in vivo results were consistent with the in vitro finding that circPOSTN promoted GBM neovascularization through upregulating STC1 expression.

### The expressions of circPOSTN and STC1 show a significant correlation in clinical GBM specimens

**Fig. 8:**
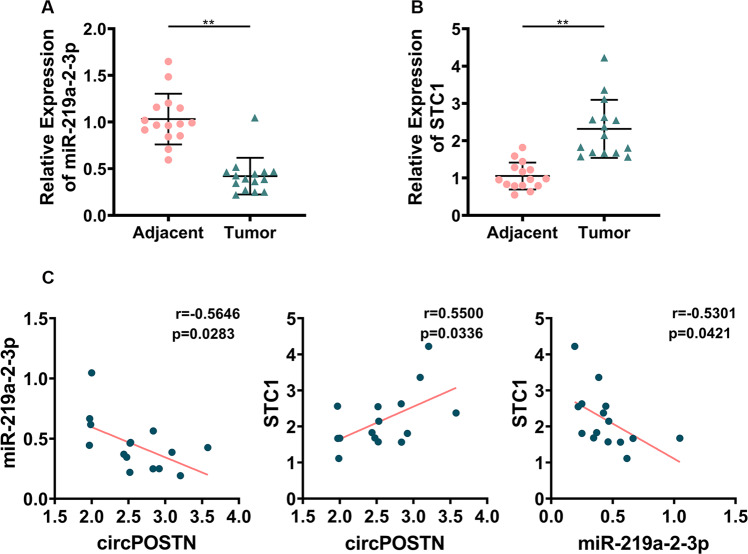
CircPOSTN sponges miR-219a-2-3p and upregulates STC1 expression in GBM clinical samples. The expression level of miR-219a-2-3p (**A**) and STC1 (**B**) were compared by quantitative RT-PCR in 15 pairs of GBM tissues and the corresponding adjacent normal tissues. **C** Pearson’s correlation analysis between the circPOSTN and miR-219a-2-3p (left panel; Pearson’s r = −0.5646), circPOSTN and STC1 (center panel, Pearson’s r = 0.5500), and miR-219a-2-3p and STC1 (right panel, Pearson’s r = −0.5301) in 15 GBM clinical samples. Data are presented as the mean ± SD. **P < 0.01.

To confirm the circPOSTN/miR-219a-2-3p/STC1 axis in GBM cells, we measured the expression levels of circPOSTN, miR-219a-2-3p, and STC1 in 15 pairs GBM and the corresponding adjacent normal specimens using quantitative RT-PCR, respectively. Initially, we found significant downregulation of miR-219a-2-3p and elevated STC1 mRNA level in GBM specimens compared to the adjacent normal tissues (Fig. 8 A, B, all *P* < 0.01). Next, based on miR-219a-2-3p, STC1, and circPOSTIN expression detected by quantitative RT-PCR, we assessed the expression correlation in the 15 pairs of specimens. Expectedly, the expression of miR-219a-2-3p presented negative correlation with circPOSTN (Person’s r = −0.5646, *P* < 0.05), while the expression of STC1 showed significantly positive co-expression with circPOSTN (Person’s r = 0.5500, *P* < 0.05). Meanwhile, the expression of STC1 became downregulated with higher miR-219a-2-3p expression (Fig. 8C).

## Discussion

Non-coding RNA is referred to the RNA transcripts that exert its function without translating into proteins, including tRNA, rRNA, small nuclear RNA (snRNA), microRNA, long non-coding RNA, and circular RNA (circRNA) [18]. CircRNA is a kind of closed circular RNA molecule initially identified by HL Sanger et al. in the 1970s [19]. Due to its special structure, circRNA becomes not easily degraded by exonucleases and therefore is more stable than linear RNA [15]. Most circRNAs are circularized from exons, while some circRNAs are lasso structures circularized from introns [20]. Initially, circRNA was considered to be invalid RNA produced by the wrong splicing of transcripts. In recent years, with the deepening of biological research, circRNA has been found to be pivotal in human diseases, including cardiovascular disease [21], renal diseases [22], fibrosis disease [23], Alzheimer’s disease [24], and especially neoplastic processes [25]. To this date, the dysregulation of circRNA has been proved to play an important primary role in tumorigenesis, including GBM. For example, the circulation progress of circMMP9 is typically induced by eukaryotic initiation factor 4A3 (eIF4A3), which boosts the overexpression of circMMP9 in GBM. Further, circMMP9 acts as a ceRNA to absorb miR-124 and ultimately promotes GBM cell proliferation, migration, and invasion [26]. As a tumor-suppressing circRNA, circ CDR1as directly interacts with p53 proteins and protects it from ubiquitin-proteasome mediated degradation, which maintains the function of repairing DNA damage and inhibits glioma tumorigenesis [27]. These studies conclusively suggest that focusing on circRNA in GBM is important in future pathogenetic studies of GBM.

Due to a larger number of miRNA response elements (MREs), circRNA is able to form the catalytic core of RNA-induced silencing complex (RISC), and therefore it can serve as a ceRNA to sponge miRNAs and limits their biological functions [16]. The ceRNA binding function of non-coding RNA was initially discovered when people used miRNA sponges to absorb miRNAs and recognized them as miRNA inhibitors [28]. Follow-up studies in plant and animal cells found that the miRNA crosstalk phenomenon was prevalent in cells, where lncRNAs and circRNAs were proved to carry MREs and be involved in the ceRNA network [16, 29, 30]. Typically, the deregulate of ceRNA networks in human cells would cause human diseases, including carcinogenesis [31]. On our hand, by using the online algorithms to predict the ceRNA network, we hypnotized that circPOSTN might fulfill a ceRNA role in GBM. RIP assay, RNA pulldown assay, and dual-luciferase reporter assay further confirmed that circPOSTN sponged miR-219a-2-3p, restored its tumor-suppressing function, and therefore upregulated the downstream gene STC1.

Stanniocalcin 1 (STC1) belongs to a kind of secreted glycoprotein with 56 kDa that is involved in the transportation progress of calcium and phosphate in the kidney and intestine [32]. STC1 was also proved to be essential in cell metabolism and cellular calcium/phosphate homeostasis [33]. Human STC1 is widely expressed in a variety of tissues and participates with cellular immortalization [34]. An increasing body of evidence suggests that the dysregulation of STC1 was strongly correlated with multiple physiological and pathophysiological processes, including cancer [35, 36]. Further studies demonstrated that STC1 is differently expressed and serves as a tumor-promoting role in various types of cancers, including breast cancer [37], ovarian cancer [38], cervical cancer [39], and gastric cancer [40]. STC1 becomes a known oncogene in breast cancer. On the one hand, high STC1 expression was correlated with poor clinical outcomes in breast cancer [41, 42]. On the other hand, STC1 promotes breast cancer proliferation and invasion by activating the JNK/c-Jun signaling pathway [43]. The dysregulation of STC1 in under a complex variety of regulatory networks, including N(6)-methyladenosine-mediated transactivation [44], miRNA-mediated mRNA degradation [45], RelA-mediated transcription [46], and so on. In glioblastoma (GBM), available studies indicate that STC1 accounts for the invasion steps of GBM [47]. More importantly, the expression level of STC1 is regulated by a given miRNA network [48]. The above shreds of evidence indicated that STC1 is a key regulator of tumorigenesis and tumor progression. In this study, by predicting the target sites on STC1 and experimental validation, we innovatively uncover a circPOSTN-medicated regulatory axis to enhance STC1 expression. Our results provide an important addition to the existing studies.

This study still has several limitations. We only performed bulk analysis of circPOSTN and STC1 in GBM tissues, while ignored the non-malignant cellular component. As the field of tumor study has now evolved to incorporate the tumor microenvironment. Our precent study has separated the expression level of circPOSTN from mixed GBM tissues, which simplifies the nature of the pathology and disregards an important element of its biology. The detail mechanisms still need to be explored in the future study.

In this study, we explore for the first time the biological function of circPOSTN in GBM. We observed an enhanced circPOSTN expression in GBM clinical samples and cell lines. Functional experiments revealed that circPOSTN promoted GBM tumor formation through increasing neovascularization. Molecular mechanism investigation revealed that circPOSTN exerted its tumor-promoting effects by acting as a ceRNA to sponge miR-219a-2-3p and therefore upregulating STC1 and VEGFA expressions.

## Materials and methods

### Cell lines and patient tissues

The human glioma cell lines (U251, LN229, U87, A172, U118, and H4), one normal human astrocyte cell line (NHA), and one normal human glial cell line (HEB) were used in this study. The human glioma cell lines and control cell lines were cultured in the Dulbecco’s Modified Eagle’s Medium (DMEM; Invitrogen, Carlsbad, CA) supplemented with 10% fetal bovine serum (FBS; Gibco, Waltham, MA) and 1% Pen/Strep (Invitrogen). All cell lines were incubated at 37 °C in a 5% CO_2_ incubator.

The consent for human tissues studies was approved by the Affiliated Hospital of Guizhou Medical University. The human glioblastoma tissues and the corresponding adjacent tissues were obtained from 15 glioblastoma patients. All patients had signed the written informed consent before we collected the tissues for research purposes. After surgical removal, the GBM tissues were quickly snap-frozen in liquid nitrogen and saved in the −80 °C fridges for further research.

### RNA extraction and quantitative reverse transcription PCR (RT-qPCR)

Total RNAs from the cell lines and human GBM tissues were extracted with TRIzol Reagent under the guidance of the protocol. The RNA quality and concentrations were determined by Nano-Drop 2000 (ThermoFisher Scientific, San Jose, CA). The relative expression levels of circPOSTN, hsa-miR-219a-2-3p, and STC1 were assessed by RT-qPCR. For circPOSTN and STC1, 2000 ng of total RNA was used for reverse transcription to cDNA with the PrimeScript™ RT Master Mix (Takara, Tokyo, Japan). The cDNA products were amplified using the TB Green® Fast qPCR Mix (Takara) according to the manufacture’s instruction. The GAPDH was used as endogenous control. For miR-219a-2-3p, the quantification was performed using the Bulge-Loop™ miRNA qPCR system (RiboBio, Guangzhou, China). The small nuclear RNA U6 was used as the internal reference gene. All primers were designed and synthesized by RiboBio Company. The relative expression levels of these genes were calculated using the 2^-∆∆Ct^ method. The primers were listed in the Supplementary Table 1.

### Fluorescent in situ hybridization (FISH)

The FISH assay was performed to visualize the subcellular location of circPOSTN using the Ribo^TM^ Fluorescent in situ Hybridization Kit (RiboBio) according to the manuscript’s guidance. Briefly, the U87 and U251 cells were seeded in the round glass slide and incubated until cells reached a confluency of 60%~70%. After washing with 1 × PBS, the cell slides were fixed with 4% paraformaldehyde buffer and permeabilized with the membrane-breaking solution. Then, the cell slides were hybridized with 2.5 μl FISH probes diluted in 200 μl of hybridization buffer at 37 °C overnight. After removal of the un-hybridized probes with SSC buffer, the cell slides were stained with DAPI buffer and sent for visualization under confocal laser microscopy. The FISH probes targeting circPOSTN were designed and synthesized by the RiboBio company. DAPI was used to stained the nuclei.

### Extraction of subcellular fractions

The subcellular fractions from GBM cells were extracted using the Nuclear/Cytosol Fractionation Kit (BioVision, MB, USA) according to the manufacture’s instruction. The GBM cells were washed twice with ice-cold 1 × PBS and lysed in 200 μl CEB-A Mix with DTT and proteinase inhibitors. After the vortex, the cell lysate was added with 11 µl ice-cold Cytosol Extraction Buffer-B and sent for centrifugation at 4 °C. The supernatant was transferred to a new tube and saved as the cytoplasmic extract. The pellet containing the nuclei was incubated with 100 µl Nuclear Extraction Buffer Mix and then vortexed to extract the nuclear lysate. Both cellular lysate and nuclear lysate were added with TRIzol buffer and sent for RNA extraction.

### Plasmid constructs, miRNA inhibitors/mimic design, and cell transfection

The overexpression vector pcDNA3.1-circPOSTN, pcDNA3.1-STC1, and the corresponding empty vector control were all designed and purchased from GENESEED Biological Company (Guangzhou, China). The miR-219a-2-3p inhibitors/mimic were designed and commercial synthesis by RiboBio Company (Guangzhou). The scrambled control inhibitors and mimic were used as the negative control.

For cell transfection, the Lipofectamine 3000 reagent was used (Invitrogen). The U87 and U251 cells were seeded in the six-well plate and incubated to reach a 60%~70% confluence. Then 2–5 μg of circPOSTN or STC1 overexpression vectors were transfected. For miR-231-2-3p inhibitors or mimic transfections, 50 nM final concentration inhibitors/mimic were transfected. Three days after transfection, the cells were submitted for RNA extraction, and RT-qPCR was used to determine the interference efficiency.

### EdU incorporation assay

The Edu incorporation assay was used to detect the proliferation ability of GBM cells using the BeyoClick™ EdU-488 Cell Proliferation Detection Kit (BeyoTime, Shanghai, China). The GBM cells were seeded in the six-well plates and incubated for adherent conditions. Then, the 2 × EdU solution was added into the culture medium to reach a 10 μM EdU final concentration. After incubation at 37 °C for 2 h, the cells were fixed with 4% paraformaldehyde, permeabilized with PBS buffer containing 0.3% Triton X-100, and stained with Click Additive Solution. The nucleic was stained with DAPI solution. The images were captured using a fluorescence microscope.

### Clone formation assay

The reproduction ability of GBM cells was assessed by clone formation assay. In general, GBM cells after transfection were trypsinized, and the cell concentration was adjusted to 1 × 10^4^ cells per ml. Then, GBM cells were seeded in the six-well plates at the density of 600 cells/well. After a 10-day incubation, the cells were grown to be clonal. Then, the clones were fixed with 4% paraformaldehyde and stained with hematoxylin. The clone number of each well were counted and recorded.

### MTT assay

The proliferation ability of GBM cells was determined by the MTT assay. In brief, after transfection, the GBM cells were resuspended and adjusted to a concentration of 1 × 10^4^ cells per ml. Then, cells were seeded into the 96-well plate at a density of 1000 cells/well (100 μl in total). After a 48-h incubation, 10 μl of MTT solution (Sigma-Aldrich) was added and incubated at 37 °C for 4 h. Then, the cells were sent for measuring the absorbance at OD 570 nm using a microplate reader. The MTT assay was duplicated six times.

### Transwell migration assay

The transwell migration assay was performed to evaluate cell migration ability using the 24-well transwell chambers (Corning, NY, USA). The GBM cells were trypsinized and resuspended in the serum-free culture medium (1 × 10^5^ cells/well). Then cells were added to the upper chamber, and the lower chamber was filled with 500 μl of culture medium containing 10% FBS. After a 16-h incubation, the GBM cells at the bottom of the chamber were fixed with 4% paraformaldehyde solution and stained with hematoxylin. After removing the non-specifically stained cells, the GBM cells that have migrated to the bottom of the chambers were observed under an inverted microscope and counted.

### Tubule formation assay

The angiogenesis ability of GBM cells was evaluated by the tubule formation assay. Briefly, 200 μl of pre-chilled Matrigel (Corning) was added in advance in a 24-well plate and polymerized at 37 °C for 30 min. 2 × 10^4^ HUVECs in 200 μl conditioned medium collected from GBM cells were seeded in each well and incubated at 37 °C for one day. The final capillary structure was captured under a ×100 inverted microscope, and the branch points and the number of tubes were recorded by ImageProPlus software.

### RNA pulldown assay

The RNA pulldown assay was used to identify the proteins interacting with circPOSTN. The pcDNA3.1-circPOSTN plasmid was linearized with single enzyme digestion, and the liner DNA templates were submitted to conduct in vitro transcript, and biotin labeling using Ribo^TM^ RNAmax-T7 Biotin Labeled Transcription Kit (RiboBio). The biotinylated circPOSTN RNAs were incubated with cell lysates extracted with RIPA lysis buffer (Beyotime) containing proteinase inhibitors and RNase inhibitors (ThermoFisher) at room temperature for 1 h. Then, the mixture was incubated with Dynabeads Streptavidin (ThermoFisher) on a rotating bracket overnight at 4 °C. Finally, after washing away the non-specifically bound substances, the proteins interacting with circPOSTN were separated from the beads and sent for conducting further analysis.

### Dual-luciferase reporter assay

The relative luciferase activity was measured using a dual-luciferase reporter system (Promega). The target sites of miR-231-2-3p to the circPOSTN and STC1 were subclone to the pmir-GLO vectors. The mutagenesis was used as non-target control. The GBM cells were seeded in a 24-well plate and co-transfected with the luciferase vectors accompanied with the miR-231-2-3p mimic/inhibitors or the negative control. After 72-h culture, the cells were lysed with lysis buffer and submitted to measure the relative luciferase activities, which were defined as the *Firefly* luciferase normalized to *Renilla* luciferase.

### RNA immunoprecipitation (RIP)

The RIP assay was conducted to verify the interaction of AGO2 proteins with circPOSTN or STC1 RNAs using the EZ-Magna RIP^TM^ Kit (Millipore, MA, USA) under the manufacture’s instruction. 2 × 10^7^ GBM cells were lysed in RIP lysis buffer and incubated magnetic beads conjugated to 5 μg anti-AGO2 antibodies (Abcam) or 5 μg normal anti-IgG antibodies at 4 °C overnight to capture the RNAs bound to AGO2 proteins. Then, the captured RNAs were extracted from the beads and submitted to qPCR analysis.

### Western blotting analysis

The western blotting analysis was used to evaluate the expression levels of target proteins in GBM tissues and cell lines. In brief, the tissues or cells were lysed in RIPA lysis buffer containing proteinase inhibitor Cocktails, and phosphorylase inhibitors. The protein concentrations were determined by BAC assay using the BCA Protein Quantification Kit (ThermoFisher). The protein solution (20 μg total proteins) was submitted to SDS-PAGE electrophoresis and transferred to NC membranes (Millipore) for 1.5 h. The membranes were blocked with 5% non-fat milk for 1 h at room temperature and then incubated with primary antibodies at 4 °C overnight and secondary antibodies at room temperature for 1 h. The membranes were visualized with Immobilon Western Chemiluminescent HRP Substrate (Millipore).

### Subcutaneous tumor model

The ethical approval for the animal study was granted by the Affiliated Hospital of Guizhou Medical University. For the subcutaneous tumor mice model, A total of 40 male BALB/C nude mice were purchased from the Guangdong Medical Laboratory Animal Center. After a one-week quarantine period, the mice were randomly divided into eight groups (five mice per group). 1 × 10^6^ U87 or U251 GBM cells in 150 µl culture medium containing 10% Matrigel were subcutaneously injected into the nude mice. Seven days after injection, the tumors appeared grossly visible,and the tumor sizes were recorded every week. On day 30, the mice were sacrificed, and the tumors were stripped and collected for further analysis.

### Immunohistochemistry (IHC) staining

The tumors from the mice model were embedded in paraffin and sectioned. The sections were deparaffinized, hydrated, and underwent antigen retrieval. Then, the sections were blocked with IHC blocking buffer containing 5% bovine serum albumin (BSA) and incubated with primary antibodies at 4 °C overnight. The next day, the sections were incubated with biotin-conjugated secondary antibodies and streptavidin-horseradish peroxidase. The sections were stained with hematoxylin and visualized under an inverted microscope.

### Statistical analysis

The quantitative data were presented as the mean ± SD. One-way ANOVA, Student’s *t* test, chi-square test, and Fischer’s test were chosen when appropriate. The statistical analysis was performed using SPSS software. For Pearson correlation analysis, the |*R* | ≥ 0.35 and *P* < 0.05 were considered a good correlation between variables. A *P* value of <0.05 was considered statistical significance in all cases. The GSEA was performed using an online GSEA tool (http://www.broadinstitute.org/gsea).

## Supplementary information

Supplementary Table and Supplementary Figure

WB original data

supplementary file 1 GBM_rnaseq_clinical

supplementary file 2 rnaseq_STC1

## Data Availability

All data generated or analyzed during this study are included in this article.

## References

[1] Andreatta F, Beccaceci G, Fortuna N, Celotti M, De Felice D, Lorenzoni M (2020). The organoid era permits the development of new applications to study glioblastoma. Cancers.

[2] Delgado-López PD, Corrales-García EM (2016). Survival in glioblastoma: a review on the impact of treatment modalities. Clin Transl Oncol.

[3] Jin J, Choi SH, Lee JE, Joo JD, Han JH, Park SY (2017). Antitumor activity of 7-O-succinyl macrolactin A tromethamine salt in the mouse glioma model. Oncol Lett.

[4] Yu H, Ding J, Zhu H, Jing Y, Zhou H, Tian H (2020). LOXL1 confers antiapoptosis and promotes gliomagenesis through stabilizing BAG2. Cell Death Differ.

[5] Agnihotri S, Zadeh G (2016). Metabolic reprogramming in glioblastoma: the influence of cancer metabolism on epigenetics and unanswered questions. Neuro Oncol.

[6] Zhou S, Wei J, Wang Y, Liu X (2020). Cisplatin resistance-associated circRNA_101237 serves as a prognostic biomarker in hepatocellular carcinoma. Exp Ther Med.

[7] Chen LL (2020). The expanding regulatory mechanisms and cellular functions of circular RNAs. Nat Rev Mol Cell Biol.

[8] Beermann J, Piccoli MT, Viereck J, Thum T (2016). Non-coding RNAs in development and disease: background, mechanisms, and therapeutic approaches. Physiol Rev.

[9] Meng S, Zhou H, Feng Z, Xu Z, Tang Y, Li P (2017). CircRNA: functions and properties of a novel potential biomarker for cancer. Mol Cancer.

[10] Han B, Chao J, Yao H (2018). Circular RNA and its mechanisms in disease: from the bench to the clinic. Pharm Ther.

[11] Li R, Jiang J, Shi H, Qian H, Zhang X, Xu W (2020). CircRNA: a rising star in gastric cancer. Cell Mol Life Sci.

[12] Chaichian S, Shafabakhsh R, Mirhashemi SM, Moazzami B, Asemi Z (2020). Circular RNAs: a novel biomarker for cervical cancer. J Cell Physiol.

[13] Chen A, Zhong L, Ju K, Lu T, Lv J, Cao H (2020). Plasmatic circRNA predicting the occurrence of human glioblastoma. Cancer Manag Res.

[14] Yang Y, Gao X, Zhang M, Yan S, Sun C, Xiao F (2018). Novel Role of FBXW7 Circular RNA in Repressing Glioma Tumorigenesis. J Natl Cancer Inst.

[15] Kristensen LS, Andersen MS, Stagsted LVW, Ebbesen KK, Hansen TB, Kjems J (2019). The biogenesis, biology and characterization of circular RNAs. Nat Rev Genet.

[16] Tay Y, Rinn J, Pandolfi PP (2014). The multilayered complexity of ceRNA crosstalk and competition. Nature.

[17] Witkos TM, Koscianska E, Krzyzosiak WJ (2011). Practical aspects of microRNA target prediction. Curr Mol Med.

[18] Quinn JJ, Chang HY (2016). Unique features of long non-coding RNA biogenesis and function. Nat Rev Genet.

[19] Sanger HL, Klotz G, Riesner D, Gross HJ, Kleinschmidt AK (1976). Viroids are single-stranded covalently closed circular RNA molecules existing as highly base-paired rod-like structures. Proc Natl Acad Sci USA.

[20] Pervouchine DD (2019). Circular exonic RNAs: when RNA structure meets topology. Biochim Biophys Acta Gene Regul Mech.

[21] Altesha MA, Ni T, Khan A, Liu K, Zheng X (2019). Circular RNA in cardiovascular disease. J Cell Physiol.

[22] Jin J, Sun H, Shi C, Yang H, Wu Y, Li W (2020). Circular RNA in renal diseases. J Cell Mol Med.

[23] Xu M, Xie F, Tang X, Wang T, Wang S (2020). Insights into the role of circular RNA in macrophage activation and fibrosis disease. Pharm Res.

[24] Mo D, Li X, Raabe CA, Rozhdestvensky TS, Skryabin BV, Brosius J (2020). Circular RNA encoded amyloid beta peptides-A novel putative player in Alzheimer’s disease. Cells.

[25] Zhou WY, Cai ZR, Liu J, Wang DS, Ju HQ, Xu RH (2020). Circular RNA: metabolism, functions and interactions with proteins. Mol Cancer.

[26] Wang R, Zhang S, Chen X, Li N, Li J, Jia R (2018). EIF4A3-induced circular RNA MMP9 (circMMP9) acts as a sponge of miR-124 and promotes glioblastoma multiforme cell tumorigenesis. Mol Cancer.

[27] Lou J, Hao Y, Lin K, Lyu Y, Chen M, Wang H (2020). Circular RNA CDR1as disrupts the p53/MDM2 complex to inhibit Gliomagenesis. Mol Cancer.

[28] Ebert MS, Neilson JR, Sharp PA (2007). MicroRNA sponges: competitive inhibitors of small RNAs in mammalian cells. Nat Methods.

[29] Kristensen LS, Hansen TB, Venø MT, Kjems J (2018). Circular RNAs in cancer: opportunities and challenges in the field. Oncogene.

[30] Franco-Zorrilla JM, Valli A, Todesco M, Mateos I, Puga MI, Rubio-Somoza I (2007). Target mimicry provides a new mechanism for regulation of microRNA activity. Nat Genet.

[31] Qi X, Zhang DH, Wu N, Xiao JH, Wang X, Ma W (2015). ceRNA in cancer: possible functions and clinical implications. J Med Genet.

[32] Yeung BH, Law AY, Wong CK (2012). Evolution and roles of stanniocalcin. Mol Cell Endocrinol.

[33] Yoshiko Y, Aubin JE (2004). Stanniocalcin 1 as a pleiotropic factor in mammals. Peptides.

[34] Chang AC, Janosi J, Hulsbeek M, de Jong D, Jeffrey KJ, Noble JR (1995). A novel human cDNA highly homologous to the fish hormone stanniocalcin. Mol Cell Endocrinol.

[35] Olsen HS, Cepeda MA, Zhang QQ, Rosen CA, Vozzolo BL, Wagner GF (1996). Human stanniocalcin: a possible hormonal regulator of mineral metabolism. Proc Natl Acad Sci USA.

[36] Chang AC, Jellinek DA, Reddel RR (2003). Mammalian stanniocalcins and cancer. Endocr Relat Cancer.

[37] Chen F, Zhang Z, Pu F (2019). Role of stanniocalcin-1 in breast cancer. Oncol Lett.

[38] Zhang C, Wang B, Wang X, Sheng X, Cui Y (2019). Sevoflurane inhibits the progression of ovarian cancer through down-regulating stanniocalcin 1 (STC1). Cancer Cell Int.

[39] Pan X, Jiang B, Liu J, Ding J, Li Y, Sun R (2017). STC1 promotes cell apoptosis via NF-κB phospho-P65 Ser536 in cervical cancer cells. Oncotarget.

[40] Yu S, Hu C, Cai L, Du X, Lin F, Yu Q (2020). Seven-gene signature based on glycolysis is closely related to the prognosis and tumor immune infiltration of patients with gastric cancer. Front Oncol.

[41] McCudden CR, Majewski A, Chakrabarti S, Wagner GF (2004). Co-localization of stanniocalcin-1 ligand and receptor in human breast carcinomas. Mol Cell Endocrinol.

[42] Zandberga E, Zayakin P, Ābols A, Pūpola D, Trapencieris P, Linē A (2017). Depletion of carbonic anhydrase IX abrogates hypoxia-induced overexpression of stanniocalcin-1 in triple negative breast cancer cells. Cancer Biol Ther.

[43] Han J, Jeon M, Shin I, Kim S (2016). Elevated STC‑1 augments the invasiveness of triple‑negative breast cancer cells through activation of the JNK/c‑Jun signaling pathway. Oncol Rep.

[44] Peng F, Xu J, Cui B, Liang Q, Zeng S, He B (2021). Oncogenic AURKA-enhanced N(6)-methyladenosine modification increases DROSHA mRNA stability to transactivate STC1 in breast cancer stem-like cells. Cell Res.

[45] Luan C, Li Y, Liu Z, Zhao C (2020). Long noncoding RNA MALAT1 promotes the development of colon cancer by regulating miR-101-3p/STC1 axis. Onco Targets Ther.

[46] Li H, Li Q, Lian J, Chu Y, Fang K, Xu A (2020). MLL2 promotes cancer cell lymph node metastasis by interacting with RelA and facilitating STC1 transcription. Cell Signal.

[47] Li Y, He ZC, Zhang XN, Liu Q, Chen C, Zhu Z (2018). Stanniocalcin-1 augments stem-like traits of glioblastoma cells through binding and activating NOTCH1. Cancer Lett.

[48] Sakata J, Sasayama T, Tanaka K, Nagashima H, Nakada M, Tanaka H (2019). MicroRNA regulating stanniocalcin-1 is a metastasis and dissemination promoting factor in glioblastoma. J Neurooncol.

