# Supplementary material for: Circular RNA circPOSTN promotes neovascularization by regulating miR-219a-2-3p/STC1 axis and stimulating the secretion of VEGFA in glioblastoma
Source: Cell Death Discov. 2022 Aug 4;8:349. doi: 10.1038/s41420-022-01136-9 (PMC9352789; doi:10.1038/s41420-022-01136-9)
Supplement: Supplementary file 1 — Supplementary Table and Supplementary Figure [file 41420_2022_1136_MOESM1_ESM.docx]

**Supplementary Table 1. Sequences of PCR primers used in this study**

| GAPDH | Forward(5’-3’) | TGTGGGCATCAATGGATTTGG |
| --- | --- | --- |
|  | Reverse(5’-3’) | ACACCATGTATTCCGGGTCAAT |
| miR-219a-2-3p | Forward(5’-3’) | GTCCAGAATTGTGGCTGGAC |
|  | Reverse(5’-3’) | GCAGGGTCCGAGGTATTC |
| circPOSTN | Forward(5’-3’) | GTTTGGACTTGGGAACAGGA |
|  | Reverse(5’-3’) | CACCATTTGTTGCAATCTGG |
| STC1 | Forward(5’-3’) | AGCCTACTGGACTGTGACGAAGAC |
|  | Reverse(5’-3’) | CTCATTGGCACGCCTCCTGTTG |

**Supplementary Figure S1. Detection of miR-219a-2-3p interference efficiency.**

**A, B.** The expression level of miR-219a-2-3p was measured by quantitative RT-PCR in U87 and U251 cells transfected with miR-219a-2-3p inhibitor (**A**) or mimic (**B**). Data are presented as the mean ± SD. ****P* < 0.001.


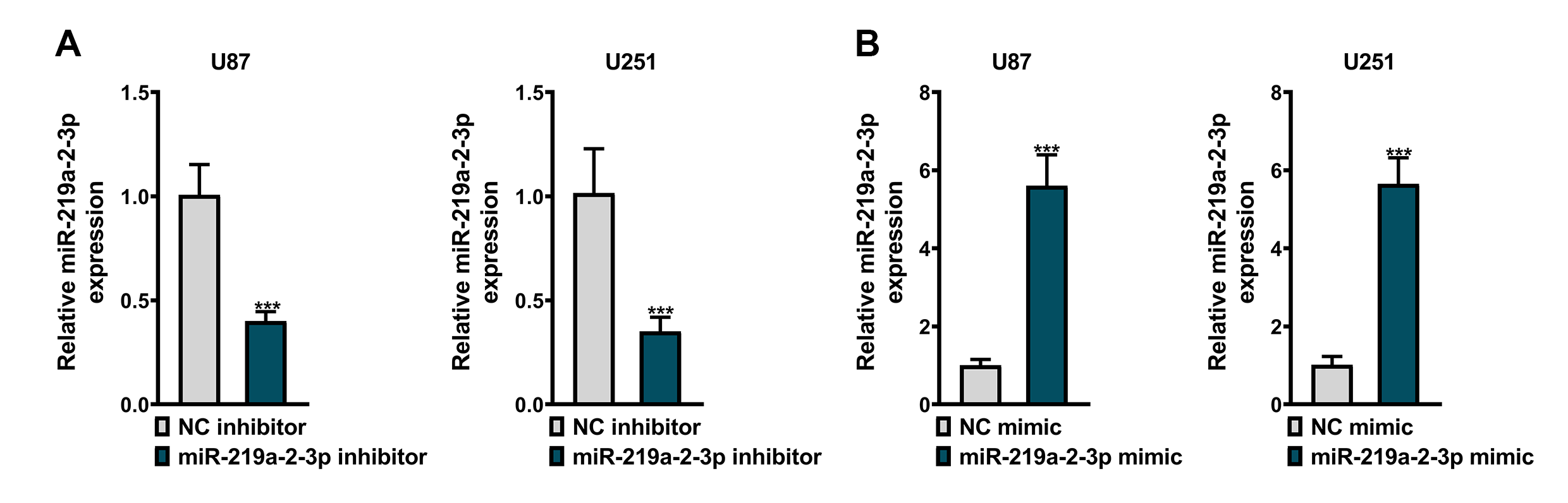


**Supplementary Figure S2. Quantitative RT-PCR and western blotting analysis were used to verify the interference efficiency of STC1 in U87 and U251 cells.**

**
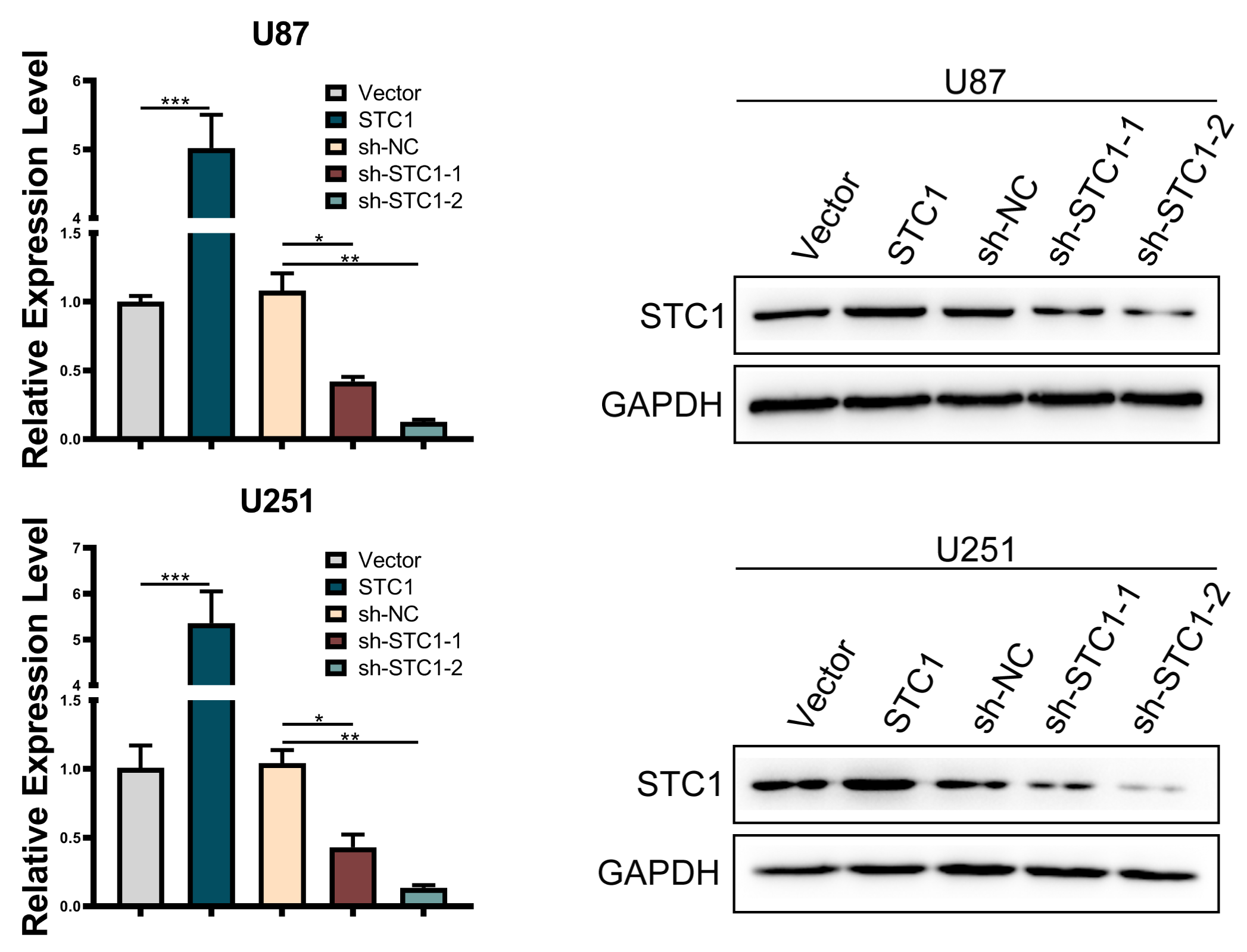
**
