# Supplementary figures and images for: Circular RNA circPOSTN promotes neovascularization by regulating miR-219a-2-3p/STC1 axis and stimulating the secretion of VEGFA in glioblastoma
Source: Cell Death Discov. 2022 Aug 4;8:349. doi: 10.1038/s41420-022-01136-9 (PMC9352789; doi:10.1038/s41420-022-01136-9)

F4A

U87

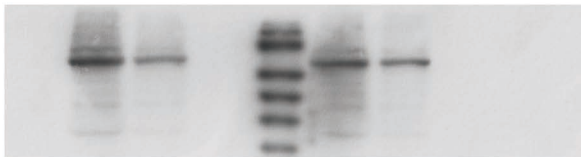

U251

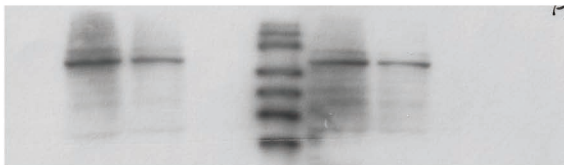

5F

STC1

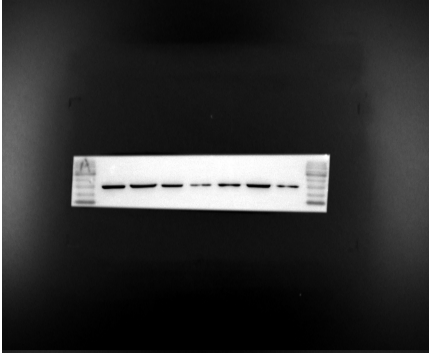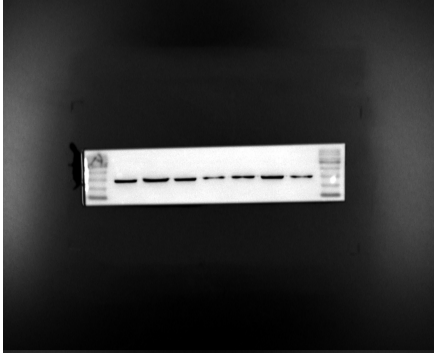

GAPDH

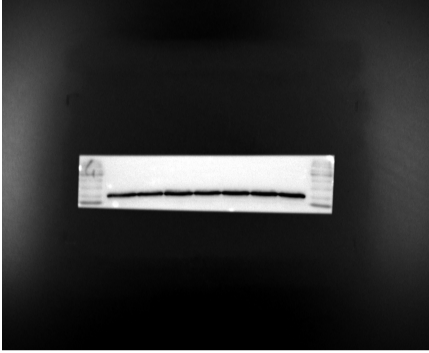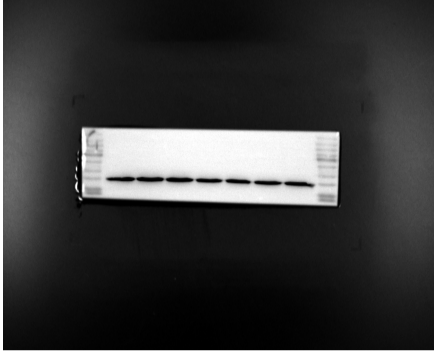

U87

U251

7D

STC1

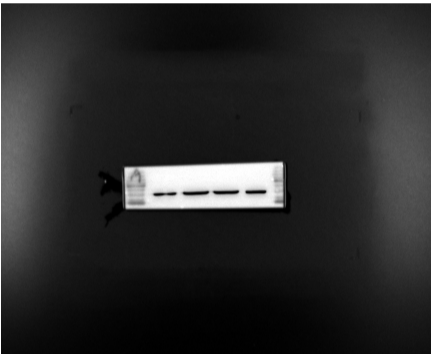

VEGF

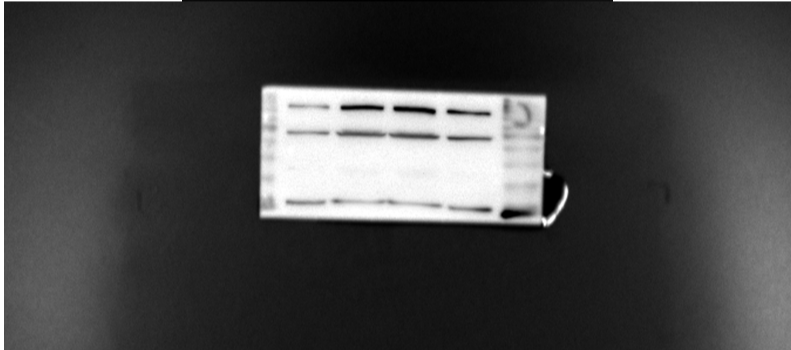

GAPDH

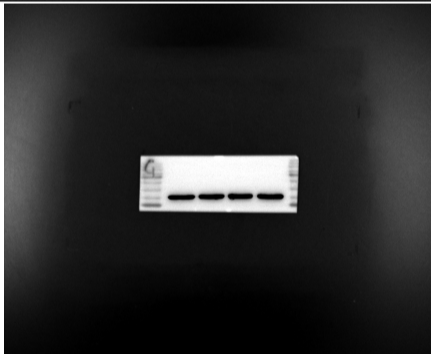

S2

STC1

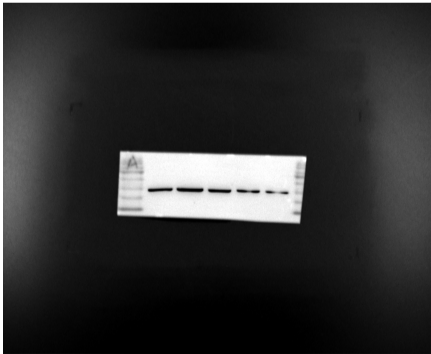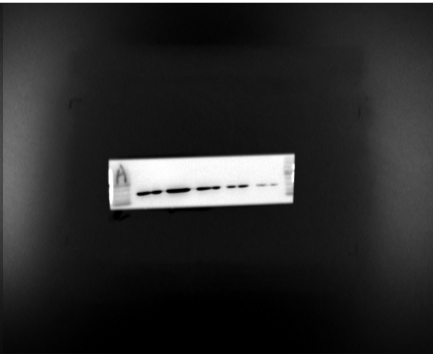

GAPDH

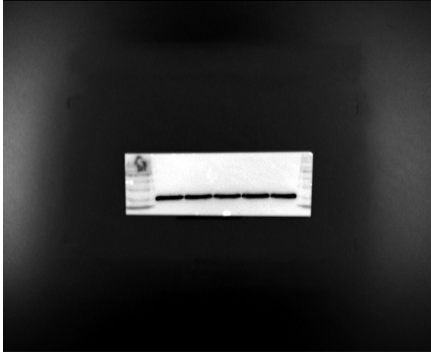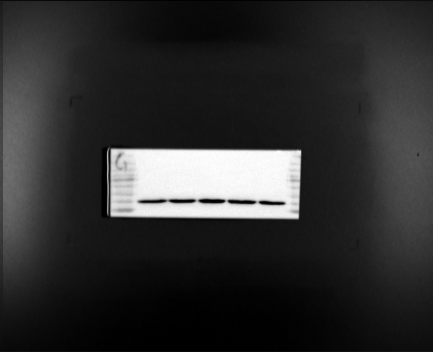

U87

U251

Supplement: Supplementary file 2 — WB original data [file 41420_2022_1136_MOESM2_ESM.pdf]
